# CoMET: A Mesquite package for comparing models of continuous character evolution on phylogenies

**Published:** 2007-02-07

**Authors:** Chunghau Lee, Sigal Blay, Arne Ø. Mooers, Ambuj Singh, Todd H. Oakley

**Affiliations:** 1 Department of Computer Science;; 2 Department of Ecology Evolution and Marine Biology, University of California, Santa Barbara, CA 93106 USA; 3 Department of Biological Sciences and IRMACS, 8888 University Drive, Simon Fraser University, Burnaby, BC V5A 1S6 Canada

**Keywords:** Maximum likelihood, Brownian motion, continuous traits, phylogeny

## Abstract

Continuously varying traits such as body size or gene expression level evolve during the history of species or gene lineages. To test hypotheses about the evolution of such traits, the maximum likelihood (ML) method is often used. Here we introduce CoMET (Continuous-character Model Evaluation and Testing), which is module for Mesquite that automates likelihood computations for nine different models of trait evolution. Due to its few restrictions on input data, CoMET is applicable to testing a wide range of character evolution hypotheses. The CoMET homepage, which links to freely available software and more detailed usage instructions, is located at http://www.lifesci.ucsb.edu/eemb/labs/oakley/software/comet.htm.

## Introduction

CoMET (Continuous-character Model Evaluation and Testing) determines and compares maximum likelihood values for various different evolutionary models of character evolution. It is licensed under the Lesser GNU Public License and runs as a package for the Mesquite Project (Maddison and Maddison 2004) a free, open-source phylogenetic analysis platform with a graphical user interface.

Given experimental data and a proposed binary phylogenetic tree with branch lengths, CoMET calculates the likelihood of observing a particular set of phenotypic data under nine different evolutionary models (Mooers et al. 1999; Oakley et al. 2005), which differ in their assumptions about how evolutionary “time” is estimated. Examples of continuous character data that could be used include gene frequencies in different species (Felsenstein 1981; Felsenstein 2004), microarray expression data for a gene family (Gu 2004; Oakley et al. 2005), and body size or vocalization data for a group of taxa (Mooers and Schluter 1998; Mooers et al. 1999). These character data represent phenotypes at the tips of the phylogenetic tree. The assumed tree topologies are usually constructed from separate data, for example by comparison of nucleotide sequences. The nine models will be discussed in more detail after a brief introduction to how ML is calculated for continuous characters.

At every internal node *p* in the tree, a contrast value is calculated, representing the node’s contribution to the overall log likelihood. Restricted Brownian diffusion is the model used to calculate the likelihood of the daughter states of a parent node, as described in Equation 1 (Felsenstein 1981; Felsenstein 2004):

(1) $$contrast_{p}:=\frac{-\text{In}(b_{1}+b_{2})}{2}-\frac{{\left(s_{1}-s_{2}\right)}^{2}}{2(b_{1}+b_{2})}$$

In this equation, *b*_1_ and *b*_2_ are the lengths of branches coming from node *p* to its two daughter nodes, and *s*_1_ and *s*_2_ are the phenotypic states of the daughters. The sum of all the internal nodes’ contrast values represents the total log likelihood. Phenotypic state data, however, are available only at the terminal nodes of the tree since there is usually little or no observed information on ancestral states. Therefore, to calculate contrasts of inner nodes, CoMET infers the state of a given internal node based on that node’s two daughters. The two equations below (Felsenstein 1981; Felsenstein 2004) weigh daughter states and branch lengths to compute their parent’s state *s_p_* (Equation 2a) and an error-accommodating value to add to the parent branch length *b_p_* (Equation 3):

(2a) $$S_{p}:=\frac{\frac{s_{1}}{b_{1}}+\frac{s_{2}}{b_{2}}}{\frac{1}{b_{1}}+\frac{1}{b_{2}}}$$

(3) $$b_{p}:=b_{p}+\frac{b_{1}b_{2}}{b_{1}+b_{2}}$$

## The Trait Evolution Models

**Figure 1:**
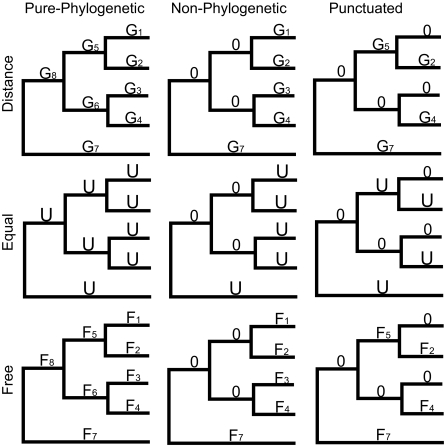
Nine different maximum likelihood models of continuous trait evolution employed in CoMET, after Oakley et al. (2005). The models predict that change in trait value increases monotonically with the “time” available for change. Time available for change is estimated in different ways for different models, as indicated by different variables above branches of a hypothetical phylogenetic tree. Branches labeled “Gi” assume trait change is equal to genetic (or other) distance of that branch. Those labeled “U” assume a unit (equal) amount of change, and those labeled “Fi” are estimated from the trait data itself (free). Branches labeled “0” assume no change in trait has occurred along that branch. Columns represent three different classes of models. The pure phylogenetic class assumes trait change occurs on every branch of the phylogeny, the non-phylogenetic class assumes trait change occurs only along terminal branches, and the punctuated class assumes trait change occurs on only one of every pair of descendent branches.

Nine models result from the combination of three different model types (distance, equal, and free) for each of three different model classes (pure-phylogenetic, non-phylogenetic, and punctuated) (Figure 1) (Oakley et al. 2005). The three model types differ in how they emphasize evolutionary rate and distance, as represented in branch lengths, when applied to the data. In the *distance models*, explicit branch lengths given to CoMET by the user represent the assumed amount of divergence in phenotype. In the *equal models,* the branch lengths are set equal to each other to represent equal divergence in phenotype between every node. In this case, only the number of bifurcations dictates the assumed amount of change in phenotype. In the *free models,* branch lengths may be any non-negative value and represent separate parameters to be estimated by maximizing the likelihood function. Each of these model types is used in three different model classes, which differ in how they emphasize branching events when modeling the data. The *pure phylogenetic class* takes the given tree topology literally and assumes phenotypic change at every branching point. The *non-phylogenetic class* is like star phylogeny by disregarding all branching points, effectively modeling close phylogenetic relatives as being no more similar to each other than to distant relatives. At every internal node in the *punctuated class,* one of daughter node retains the phenotypic state of the parent, while the other daughter node is free to vary.

For the six non-punctuated models, CoMET follows this execution pattern:

1. Copy the given tree and readjust its parameters according to the current model. For example, CoMET attempt store assign branch lengths that maximize the ML in the case of the *free model*. For models of the *non-phylogenetic class*, the lengths of every non-terminal branch are set to zero.
2. Adjust the rate of evolutionary change by scaling the whole tree with a common value (Oakley et al. 2005). Then calculate the total likelihood by computing contrasts recursively. Repeat until the ML-maximizing scalar is found.
3. Transform ML into Aikaike Information Criterion (AIC) values, normalizing data according to different degrees of freedom (Oakley et al. 2005).

## Computing the Free Models

An ML calculation problem arises for the *free models* due to the requirement for the *free model* to allow for branch lengths of zero. To avoid division by zero in Equation 2a, only one daughter branch may be zero. Therefore, for the *free models*, Equation 2a is replaced with Equation 2b, which says that if a parent node *p* has a branch with length 0 going to daughter *d,* the parent state *s_p_* must then be assigned to be *s_d_,* the state of daughter *d:*

(2b) $$S_{p}:=s_{d}|b_{d}=0$$

## Computing the Models of the Punctuated Class

A *punctuated* tree in CoMET has, at each internal node, one daughter branch with length zero and another with a non-zero length. In addition, CoMET implements two variants of the *punctuated class:* the *punctuated maximal* and the *punctuated average.* The *punctuated maximal* calculates the ML of just one tree using a greedy algorithm to choose which branch lengths to set to zero. By contrast, the *punctuated average* averages ML over all possible combinations of punctuated branch length assignments. To reduce the cost of calculating this average, CoMET does not actually repeat all the calculations over all the combinations. This is because among all the combinations of branch length assignments, common subtrees exist such that CoMET only needs to multiply the total contrast of just one subtree by the number of those subtrees. Consequently, the *punctuated average* calculations avoid exponential running times, as the algorithm below shows:

1. If the current node’s children are leaf nodes, sum the only two (branch length to remain non-zero or not) combinations’ contrasts and return.
2. Let *T_A_* be the subtree of daughter *A,* and *T_B_* be the subtree of daughter *B.* Let *I* and *J* be the number of internal nodes of each respective subtree.
3. Calculate *ML_A_* *and ML_B_* as the total of ML of the combinations in *TA* and *TB*, respectively. This is the recursive step.
4. Let *ML*′*_A_*: = ML_A_* 2*^J^* ^+ 1^·*T_B_* has a total of 2*^J^* assignment combinations, and the parent node *P* has two more (zero/non-zero or non-zero/zero for the left and right daughter branch lengths). Consequently, 2*^J^* ^+ 1^ represents the total number of combinations outside of *T_A_*, meaning that *ML_A_* would be added to the results from the rest of the tree 2*^J^*^+1^ times. Knowing this, CoMET multiplies *ML_A_* by 2*^J^*^+1^.
5. Likewise, *ML*′*_B_*:=*ML_B_* * 2*^I^*^+1^.
6. Efficiently compile all possible states at this node *P* and calculate the contrasts of this node. Let *ML_P_* be the sum of these contrasts.
7. The total ML at current parent node *P*, covering all combinations, is *ML*′*_P_*:= *ML_P_* + *ML*′*_A_* *+ ML*′*_B_*.
8. The average ML is then *ML*′*_P_*/*k,* where *k*:= 2*^n^*^−1^ and *n* is the number of taxa and *n*−1 is the number of internal nodes in the tree.

## Summary

CoMET calculates the likelihood of observing a set of continuously varying character data while assuming nine different models of evolution. Its main strengths include whole-tree scaling and the fast pruning algorithm for the *punctuated average class.* In addition, as a package for the Mesquite Project, it is easily accessible to the user. Future work will include simulating punctuated data to compare the *punctuated maximal* and the *punctuated average classes.*
